# Neuroinflammation in advanced canine glaucoma

**Published:** 2010-10-15

**Authors:** Bing Jiang, Matthew M. Harper, Helga Kecova, Grazyna Adamus, Randy H. Kardon, Sinisa D. Grozdanic, Markus H. Kuehn

**Affiliations:** 1 Department of Ophthalmology and Visual Sciences, University of Iowa, Iowa City, IA; 2 Department of Ophthalmology, 2nd Xiangya Hospital, Central South University, Changsha, PR China; 3 Department of Veterinary Clinical Sciences, College of Veterinary Medicine, Iowa State University, Ames, IA; 4 Department of Ophthalmology, Oregon Health and Science University, Portland, OR; 5 Iowa City Veterans Administration Center for Prevention and Treatment of Visual Loss, Iowa City, IA

## Abstract

**Purpose:**

The pathophysiological events that occur in advanced glaucoma are not well characterized. The principal purpose of this study is to characterize the gene expression changes that occur in advanced glaucoma.

**Methods:**

Retinal RNA was obtained from canine eyes with advanced glaucoma as well as from healthy eyes. Global gene expression patterns were determined using oligonucleotide microarrays and confirmed by real-time PCR. The presence of tumor necrosis factor (TNF) and its receptors was evaluated by immunolabeling. Finally, we evaluated the presence of serum autoantibodies directed against retinal epitopes using western blot analyses.

**Results:**

We identified over 500 genes with statistically significant changes in expression level in the glaucomatous retina. Decreased expression levels were detected for large number of functional groups, including synapse and synaptic transmission, cell adhesion, and calcium metabolism. Many of the molecules with decreased expression levels have been previously shown to be components of retinal ganglion cells. Genes with elevated expression in glaucoma are largely associated with inflammation, such as antigen presentation, protein degradation, and innate immunity. In contrast, expression of many other pro-inflammatory genes, such as interferons or interleukins, was not detected at abnormal levels.

**Conclusions:**

This study characterizes the molecular events that occur in the canine retina with advanced glaucoma. Our data suggest that in the dog this stage of the disease is accompanied by pronounced retinal neuroinflammation.

## Introduction

Glaucoma is among the leading causes of human blindness world wide and continues to pose a clinical challenge yet the sequence of the pathophysiological events that accompany and lead to retinal ganglion cell (RGC) death, the ultimate cause of vision loss in glaucoma, remains incompletely understood. Dogs frequently develop glaucoma spontaneously with advanced age and represent an attractive model for glaucoma research due to the size of their eye, the chronic nature of the disease, and the pathophysiological similarities to glaucoma in humans. In this species ocular exams such as gonioscopy, fundus photography, intraocular pressure (IOP) measurements, slitlamp exams, and indirect ophthalmoscopy are routinely performed and even advanced diagnostic methodologies such as optical coherence tomography, ultrasound, or pattern electroretinogram (pERG) recordings can be conducted [1,2].

An important step toward a better understanding of the pathophysiology of glaucoma is to determine the retinal gene expression profile during the progression of the disease. Several excellent studies describing changes in the global gene expression pattern in the retina and optic nerve of rodent models of glaucoma have been published previously [3-7].

Here, we examine the gene expression pattern and immune response changes of the retina in healthy eyes and in eyes of dogs with spontaneous glaucoma. Glaucomatous damage in these eyes was typically advanced, allowing insight into the cellular events that occur during late stage glaucoma.

## Methods

### Canine eyes

All studies were conducted in accordance with the ARVO Statement for the Use of Animals in Ophthalmic and Visual Research and are approved by the Iowa State University Committee on Animal Care. Before inclusion in the study all animals were evaluated by a veterinary ophthalmologist (SDG) to rule out the presence of non-related ocular disease. Examinations included slit lamp biomicroscopy, intraocular pressure measurements, indirect ophthalmoscopy, and gonioscopy.

Glaucoma eyes (n=9) were derived from the patient population of the Iowa State University College of Veterinary Medicine Clinics and enucleations were performed with the animal owner’s consent to ease pain and suffering. Retinal samples from total of five glaucomatous eyes were used for microarray analysis, while retinal samples of all nine animals were used for PCR analysis. All glaucoma donors were diagnosed with primary glaucoma based on abnormal gonioscopy examination, elevated IOP and absence of other ocular disease. IOP of affected eyes ranged from 30 to 48 mmHg. None of the glaucoma animals used in this study received surgical treatment, but all of them were treated with IOP lowering topical medications.

In addition, eyes from five control dogs without ophthalmic findings were used. These animals were euthanized for reasons unrelated to this study (see Table 1).

**Table 1:** Samples used for gene array analyses.

**Sample ID**	**Breed/Age**	**IOP (mmHg)**	**Duration**	**Gonioscopy**	**Medications**
G1	Shiba Inu, 5y	46	6 months	Closed angle	Latanoprost, brinzolomide
G2	Shiba Inu, 12y	33	8 months	Closed angle	Latanoprost, brinzolomide
G3	Dalmatian, 7y	48	1.5 months	Closed angle	Latanoprost, brinzolomide
G4	Basset Hound, 7y	36	4 months	Closed angle	Latanoprost, brinzolomide
G5	Basset Hound, 5y	30	6 months	Closed angle	Latanoprost, brinzolomide
C1	Beagle, 4y	12	n/a	Open angle	n/a
C2	Beagle, 4y	14	n/a	Open angle	n/a
C3	Beagle, 4y	12	n/a	Open angle	n/a
C4	Beagle, 4y	18	n/a	Open angle	n/a
C5	Beagle, 4y	17	n/a	Open angle	n/a

All glaucoma samples had closed irido-corneal angle appearance during gonioscopy examination, whereas control individuals had open angles and normal IOP. Four additional glaucoma samples were used for RT–PCR analyses.

### Gene expression analyses

Eyes were dissected and preserved in RNAlater (Ambion, Austin, TX) immediately after enucleation. Samples were then stored at −80 °C until RNA extraction. The neural retina was isolated and total RNA was extracted from the tissue using Qiagen RNeasy minipreps. Samples were treated with RNase free DNase and the integrity of the RNA was evaluated through analysis with a Bioanalyzer (Agilent Technologies, Foster City, CA). RNA was amplified using a T7 RNA polymerase based approach and hybridized to Affymetrix Canine genome 2.0 gene chips following standard protocols.

Raw data obtained were normalized using the RMA algorithm. Normalized data were log^2^-transformed and filtered to remove non-expressed genes from the data set. For the purpose of this study, expressed genes are defined as those with corresponding probesets displaying log-expression values above 7.0 in at least 2 samples (either controls or affected). The remaining probesets were analyzed to identify significant expression changes using the Wilcoxon unpaired rank sum test and the significance analysis for microarray (SAM; Version 3.0; Microsoft Excel Add-In; Stanford University, Palo Alto, CA). Data were analyzed four times using 200 permutations and different seeds values for the random number generator. The delta value was set at 0.53 and a minimum twofold expression change was required. Only genes identified as differentially expressed in all four analyses are presented in this manuscript.

The Database for Annotation, Visualization and Integrated Discovery (DAVID) was used to obtain current Entrez Gene IDs (November, 2007) and the corresponding gene names are used throughout this manuscript. Data have been deposited to the NCBI Gene Expression Omnibus (GEO) and are available under the accession number GSE21879.

### Quantitative PCR analyses

Total retinal RNA was extracted from retinas of nine glaucomatous and five control eyes and treated with DNase. These included all eyes used for the microarray studies as well as four additional glaucoma samples. Only one eye from each animal was used. From each sample 500 ng RNA was reverse transcribed in a random primed reaction and 5 ng was used as template in each PCR reaction. DNA amplification was monitored using the dye SYBR Green (Perkin Elmer, Waltham, MA). Data from each sample was obtained in triplicate; amplification controls included wells containing genomic DNA only and those containing no target (water controls). Transcript levels were determined based upon standard curves for each primer pair (Table 2). Melt curve analyses were performed following each amplification reaction to ascertain the absence of nonspecific amplification products. Expression values obtained were normalized to transcript levels for ubiquitin C (*UBC*).

**Table 2:** Oligonucleotide primers used for quantitative PCR analyses.

**Gene**	**Forward (5′-3′)**	**Reverse (5′-3′)**
*UBC*	TTGTTCGTCTCCGTGCGCTT	TGGATCTTCGCCTTGACGTTCT
*TNFRSF1A*	TCCAGTGCAATAACTGCAGCCT	ACAACTTCCCGCACTCTGTGTT
*TNFRSF14*	AGGGACACGATGTGTGAAGACT	AGCATGTGCTTCCCGCTGAA
*S100A1*	ACCTCAGGTCCAGGCTGACT	AGCTCCTTCTTGCTCAGCTTGT
*NTF3*	AAGAGGTACGCGGAGCATAA	TTGACAGGCCTGGCTTCTTT
*NRCAM*	ACGATGTCCCAAATCCTCCGTT	ATAGCCCTGCTTCGTGCATT
*GMFG*	TGCTGCTGCCACTGGT	TGTGGCACTTCGTACAGCAA
*CSF1R*	GTTGGTCACCTGCATGTCCATCAT	ACTCCCACTTCTCATTGTAGGGCA

### Immunohistochemistry

Tissue samples for immunohistochemistry were fixed in 4% paraformaldehyde, embedded in paraffin and sectioned to a thickness of 2 microns. Sections were deparaffinized with heat and xylene and rehydrated by serial rinses in decreasing concentrations of ethanol. Endogenous peroxidase activity was quenched by incubation with 3% H_2_O_2_ for 10 min. Following rinses in potassium phosphate-buffered saline (KPBS), cells were incubated in blocking solution containing 5% normal donkey serum (NDS, 017–000–121; Jackson ImmunoResearch, West Grove, PA), 0.1% BSA (BSA, A9647; Sigma, St. Louis, MO), and 0.04% Triton X-100 for 2 h to eliminate non-specific antibody labeling. Tissue was then incubated in primary polyclonal antibodies overnight at room temperature including: anti-glial fibrillary acid protein (1:2,000; Dako, Carpinteria, CA); anti-CD3 (1:75; Dako); anti-TNF-alpha (1:50; Abcam, Cambridge, MA), anti-TNF-R1 (1:500; Abcam) and anti-TNF R2 (1:25; Abcam). Antibody binding was visualized through incubation with appropriate biotinylated secondary antibodies followed by incubation with avidin-peroxidase conjugate and 3,3′-diaminobenzidine (DAB) with nickel sulfate. Care was taken to maintain identical development times in those cases where labeling intensity was measured. Sections were dehydrated through a graded ethanol series, cleared with xylene, and coverslipped. Negative controls were performed in parallel and included the omission of the primary or secondary antibody. Images of anti-GFAP and anti-CD3 were taken withan Axioplan 2 microscope (Carl Zeiss MicroImaging, Inc., Thornwood, NY), equipped with a color camera (AxioCam MRc; Carl Zeiss Meditec, Inc.).

### TNF and TNF receptor quantification

Four images of the central and peripheral retina were taken for each section using a Nikon Microphot Microscope (Nikon Inc. Garden City, NY) and a 40× oil immersion objective. Central retinal images were obtained within two microscope fields of the optic nerve. Peripheral retinal images were obtained 7–8 microscope fields away from the optic nerve. The microscope settings for tissue stained with a particular antibody were left consistent to eliminate variation from one sample to the next. A blank image that did not contain any tissue was obtained to correct for any slight variations in the slides. Metamorph image analysis software (Ver. 7; Molecular Devices, Sunnyvale CA) was used to quantify the percentage of the retina that was immunoreactive for each antibody. Blank images from the data set were used to correct each slide to account for differences in light illumination. A threshold two standard deviations below the median staining intensity for each group stained with an antibody was determined, the immunoreactivity was pseudocolored and the fraction of the retina labeled was calculated using Metamorph. The immunoreactivity of all retinal layers combined was quantified. Additionally, the combined ganglion cell layer and the inner plexiform layers were analyzed independently. Morphometric data were statistically analyzed using Students *t*-test and Graphpad Prism (ver. 4.0 for Macintosh; Graphpad Software, La Jolla, CA).

### Detection of autoantibodies

Initial screening of dog sera was performed using dog retinal proteins that were extracted from a dog retina with 2% octyl glucoside in phosphate/saline buffer (PBS) with proteolytic inhibitors, pH 7.2. The proteins were separated by SDS-gel electrophoresis on a 10% gel and transferred to an Immobilon membrane (Millipore, Bedford, MA). Individual strips containing 10 μg retinal proteins were blocked with 10% normal goat serum, 1% BSA in PBS for 1 h, and then incubated with 1:100 diluted dog serum (1 h) followed by a 1 h incubation with 1:1,000 diluted anti-dog IgG (H and L chain) conjugated to alkaline phosphatase (Sigma, St. Louis, MO). Color reaction was developed by adding the phosphatase substrate until dark bands, appeared in comparison to the positive controls (anti-recoverin antibody diluted 1:50,000, anti-enolase antibody diluted 1:2,000, anti-crystalline-μ antibodies diluted 1:1,000). Western Blots were run and examined in a masked fashion. As a negative control, serum was omitted and only a secondary antibody was applied.

## Results

All animals (Table 1) received an ocular exam (slitlamp biomicroscopy, intraocular pressure measurements, indirect ophthalmoscopy, and gonioscopy) to rule out the presence of the non-related ocular disease before inclusion in the study. In the majority of canine breeds glaucoma develops through a gradual narrowing and eventual closure of the angle resulting in elevation of IOP and the development of functional deficits. All glaucoma animals used in this study presented with closed irido-corneal angles during gonioscopy examination.

### Histology

**Figure 1:**
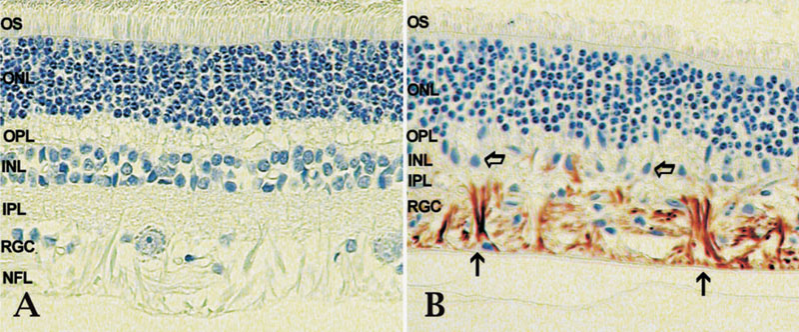
Morphology of the canine retina. Morphology of the peripheral retina in a healthy dog (**A**) and dog with advanced glaucoma (**B**). Glaucomatous changes include dramatic loss of cells in the retinal nerve fiber layer, ganglion cell layer and inner nuclear layer compared to healthy eyes. In the glaucomatous retina GFAP can readily be detected in retinal glial cells (arrows) and indicates extensive gliosis in the nerve fiber layer. GFAP can also be detected in the NFL of normal eyes when extended periods of color development are used. (NFL-nerve fiber layer, RGC-retinal ganglion cell layer, IPL-inner plexiform layer, INL-inner nuclear layer, OPL-outer plexiform layer, ONL-outer nuclear layer, OS-Photoreceptor cell outer segments).

Histological evaluation of the retina and optic nerve head (ONH) of glaucomatous canine eyes demonstrated that the morphological findings in this species closely resemble those observed in human eyes. As in human glaucoma, the appearance of the ONH in the glaucomatous eye is often characterized by extensive cupping, reorganization of the extracellular matrix and gliotic changes [2]. In the retina of dogs with advanced glaucoma a general thinning of the peripheral retina, associated with extensive loss of cells in the ganglion cell and inner nuclear layers, is evident (Figure 1). Although some loss of nuclei in the outer nuclear layer is discernable, the integrity and organization of the photoreceptor cells is largely maintained. Immunohistochemical analyses demonstrated enhanced expression of glial fibrillary acidic protein (GFAP) in the glaucomatous retina (Figure 1B). GFAP can also be detected in the nerve fiber layer of normal eyes but at much reduced levels (Figure 1A).

### Gene expression analysis

To identify retinal gene expression changes associated with advanced glaucoma, we analyzed the global gene expression patterns in five eyes derived from dogs with glaucoma and from five control animals. As observed in similar analyses of experimental models of glaucoma [3-7] our analyses indicate significant changes in the transcript levels of a large number of genes. Under the stringent parameters used here (predicted false discovery rate=0.3%) 1,111 probe sets were identified as being differentially expressed between the two groups. Of these, signals from 586 probe sets are significantly reduced in the glaucomatous retina. Further exclusion of probe sets corresponding to unannotated genes results in 362 probe sets representing 275 unique genes (Table 3). In addition, 525 probe sets are detected at increased transcript levels in the glaucomatous retina. Of these, 486 probe sets remain after removal of unannotated genes, representing 303 unique genes (Table 4).

**Table 3:** Selected genes detected at decreased levels in the glaucomatous canine retina.

**Probe Set ID**	**Gene**	**Annotation**	**Glau_avg**	**Cont_avg**	**Fold Change**	**q-value(%)**
**RGC genes**						
CfaAff × 0.30898.1.S1_at	CALB2	“calbindin 2, (calretinin)”	7.4	9.5	3.8	0
CfaAff × 0.29820.1.S1_s_at	CIRBP	cold inducible RNA binding protein	9.0	10.3	2.3	0
CfaAff × 0.27495.1.S1_at	CPLX3	complexin 3	9.1	10.5	2.3	0
Cfa.12330.1.A1_s_at	EBF1	early B-cell factor 1	5.1	7.2	4.3	0
Cfa.9181.1.A1_at	ELAVL2	“ELAV (embryonic lethal, abnormal vision)-like 2”	6.7	9.2	4.6	0
Cfa.19294.1.S1_s_at	ELAVL4	“ELAV (embryonic lethal, abnormal vision)-like 4”	6.1	9.2	6.3	0
*Cfa.299.1.A1_at	FABP3*	“fatty acid binding protein 3, muscle and heart”	7.6	9.4	2.9	0
Cfa.1153.1.A1_at	FKBP1B	“FK506 binding protein 1B, 12.6 kDa”	8.3	10.2	3.5	0
*Cfa.5865.1.A1_at	KIF5A*	kinesin family member 5A	6.7	8.5	2.8	0
Cfa.10948.1.S1_at	LOC610447	Visinin-like protein 1 (VILIP)	7.5	11.5	9.9	0
*CfaAff × 0.19019.1.S1_s_at	NEFH*	“neurofilament, heavy polypeptide 200kDa”	6.6	7.9	2.3	0
Cfa.10952.1.A1_s_at	NEFL	“neurofilament, light polypeptide 68kDa”	8.6	12.6	9.3	0
*Cfa.10905.1.A1_at	NEFM*	“neurofilament, medium polypeptide 150kDa”	8.8	12.9	10.3	0
*Cfa.11184.1.A1_s_at	NELL2*	NEL-like 2 (chicken)	6.9	8.8	3.7	0
Cfa.9043.1.A1_at	NRG1	neuregulin 1	6.3	7.4	2.2	0
CfaAff × 0.14934.1.S1_at	NRN1	neuritin 1	6.6	9.2	5.5	0
Cfa.12391.1.A1_s_at	RBPMS	RNA binding protein with multiple splicing	6.8	7.7	2.0	0
*CfaAff × 0.11569.1.S1_s_at	SCN1B*	“sodium channel, voltage-gated, type I, beta”	6.0	7.4	2.7	0
*CfaAff × 0.13307.1.S1_s_at	STMN2*	stathmin-like 2	8.9	10.4	3.1	0
*Cfa.4489.1.S1_at	UCHL1*	ubiquitin carboxyl-terminal esterase L1	11.4	12.7	2.6	0
**Synapse and Synaptic Transmission**						
*CfaAff × 0.21696.1.S1_at	ACHE*	acetylcholinesterase	6.0	7.4	2.4	0
CfaAff × 0.11508.1.S1_s_at	CADPS	Ca^2+^dependent secretion activator	8.6	10.4	3.3	0
CfaAff × 0.18352.1.S1_s_at	CPNE6	copine VI (neuronal)	6.8	7.9	2.1	0
Cfa.9427.1.A1_at	CRH	corticotropin releasing hormone	6.5	8.6	3.8	0
Cfa.21295.1.S1_s_at	DRD1IP	dopamine receptor D1 interacting protein	7.3	9.5	4.6	0
CfaAff × 0.4606.1.S1_at	GABBR2	“gamma-aminobutyric acid (GABA) B receptor, 2”	6.4	7.5	2.1	0
CfaAff × 0.26365.1.S1_at	GABRA1	“gamma-aminobutyric acid (GABA) A receptor, alpha 1”	5.4	7.7	4.3	0
*Cfa.11206.1.A1_at	GABRB3*	“gamma-aminobutyric acid (GABA) A receptor, beta 3”	6.8	9.5	5.4	0
CfaAff × 0.26360.1.S1_s_at	GABRG2	“gamma-aminobutyric acid (GABA) A receptor, gamma 2”	6.3	8.4	4.1	0
CfaAff × 0.5585.1.S1_s_at	GABRR1	“gamma-aminobutyric acid (GABA) receptor, rho 1”	6.3	8.8	5.2	0
*Cfa.1206.2.A1_s_at	GAD1*	“glutamate decarboxylase 1 (brain, 67kDa)”	5.7	8.1	4.4	0
*CfaAff × 0.13687.1.S1_at	GRIA2*	“glutamate receptor, ionotropic, AMPA 2”	7.4	9.1	3.4	0
CfaAff × 0.22871.1.S1_at	GRIA4	“glutamate receptor, ionotrophic, AMPA 4”	6.2	7.6	2.5	0
CfaAff × 0.3513.1.S1_at	GRM8	“glutamate receptor, metabotropic 8”	5.2	7.8	5.5	0
CfaAff × 0.4263.1.S1_s_at	ICA1	“islet cell autoantigen 1, 69kDa”	6.1	7.6	2.7	0
Cfa.9545.1.A1_at	NOVA1	neuro-oncological ventral antigen 1	7.0	8.2	2.3	0
CfaAff × 0.28644.1.S1_at	RAB33A	“RAB33A, member RAS oncogene family”	7.1	8.2	2.1	0
CfaAff × 0.11242.1.S1_s_at	SYNPR	synaptoporin	6.0	8.2	4.1	0
CfaAff × 0.18684.1.S1_s_at	SYT14	synaptotagmin XIV	6.5	7.8	2.6	0
CfaAff × 0.27070.1.S1_at	SYT4	synaptotagmin IV	5.5	7.3	3.7	0
Cfa.4050.1.A1_a_at	TAC1	“tachykinin, precursor 1”	6.1	8.2	3.4	0
**Phototransduction**						
CfaAff × 0.25681.1.S1_at	ARR3	“arrestin 3, retinal (X-arrestin)”	10.3	12.9	2.1	0
Cfa.3480.1.A1_s_at	GNGT2	“guanine nucleotide binding protein, gamma”	10.3	12.3	2.4	0.064323389
CfaAff × 0.3367.1.S1_at	OPN1SW	“opsin 1, short-wave-sensitive”	7.6	10.6	3.5	0.064323389
CfaAff × 0.12695.1.S1_s_at	PDE6C	cGMP-specific phosphodiesterase 6C	7.1	8.6	2.0	0.064323389
**Synucleins**						
*CfaAff × 0.15497.1.S1_s_at	SNCA*	“synuclein, alpha “	5.8	7.4	2.9	0
*CfaAff × 0.25469.1.S1_s_at	SNCB*	“synuclein, beta”	7.1	8.5	2.3	0
CfaAff × 0.24686.1.S1_at	SNCG	“synuclein, gamma”	5.9	8.5	5.1	0
**Growth Factors**						
Cfa.19396.1.S1_s_at	FGF12	fibroblast growth factor 12	5.5	7.0	2.9	0
*CfaAff × 0.9858.1.S1_s_at	FGF14*	fibroblast growth factor 14	6.8	8.0	2.2	0
*Cfa.3888.1.S1_at	IGF1*	insulin-like growth factor 1 (somatomedin C)	4.8	6.5	3.5	0
CfaAff × 0.2203.1.S1_at	IL5	“interleukin 5 (colony-stimulating factor, eosinophil)”	5.0	7.2	4.4	0
*Cfa.4064.1.A1_at	PTN*	pleiotrophin (neurite growth-promoting factor 1)	6.4	8.4	4.5	0
**Cell Adhesion**						
CfaAff × 0.14553.1.S1_at	AMIGO2	adhesion molecule with Ig-like domain 2	6.4	8.1	3.2	0
CfaAff × 0.29067.1.S1_at	CDH12	“cadherin 12, type 2 (N-cadherin 2)”	6.1	7.7	2.9	0
CfaAff × 0.29091.1.S1_at	CDH18	“cadherin 18, type 2”	5.6	7.4	3.4	0
CfaAff × 0.1039.1.S1_at	CDH7	“cadherin 7, type 2”	7.4	8.6	2.4	0
Cfa.9512.1.A1_at	CTNNA2	“catenin (cadherin-associated protein), alpha 2”	7.3	8.8	3.1	0
Cfa.3718.1.S1_s_at	DSC2	desmocollin 2	6.1	7.4	3.0	0
CfaAff × 0.13441.1.S1_s_at	EDIL3	EGF-like repeats and discoidin I-like domains 3	5.6	7.3	2.7	0
Cfa.2219.1.A1_at	ITGA6	“integrin, alpha 6”	7.5	8.3	2.2	0
*CfaAff × 0.8342.1.S1_at	PCDH9*	protocadherin 9	6.4	7.7	2.5	0
Cfa.9146.1.A1_at	PCDHAC2	“protocadherin alpha subfamily C, 2”	7.0	8.8	3.3	0
Cfa.9467.1.A1_at	RELN	reelin	8.4	9.6	2.5	0
**Calcium Binding**						
*Cfa.7840.1.A1_at	CABP5*	calcium binding protein 5	8.6	11.2	4.7	0
*CfaAff × 0.14235.1.S1_s_at	CALB1*	“calbindin 1, 28kDa”	6.3	10.0	6.8	0
Cfa.4168.2.S1_s_at	CALM1	“calmodulin 1 (phosphorylase kinase, delta)”	10.2	11.2	2.4	0
Cfa.20415.1.S1_at	HPCA	hippocalcin	7.4	8.9	2.6	0
Cfa.10160.1.S1_at	PPP3CA	“protein phosphatase 3, catalytic subunit, alpha isoform”	9.3	10.2	2.1	0
Cfa.10339.1.S1_at	PPP3R1	“protein phosphatase 3, regulatory subunit B, alpha isoform”	8.3	9.5	2.4	0
Cfa.10159.1.S1_at	PRKCB1	“protein kinase C, beta 1”	8.0	9.8	4.3	0
*Cfa.20981.1.S1_s_at	PVALB*	parvalbumin	7.7	12.1	12.0	0
**Calcium Channels**						
*Cfa.1728.1.A1_s_at	CACNA2D3*	“Ca channel, voltage-dependent, alpha 2/delta 3 subunit”	6.3	8.5	4.2	0
Cfa.1210.1.S1_at	CACNB3	“Ca channel, voltage-dependent, beta 3 subunit”	7.2	8.3	2.6	0
Cfa.269.1.A1_at	RYR2	ryanodine receptor 2	5.3	7.0	3.2	0
CfaAff × 0.7090.1.S1_s_at	TRPC3	“transient receptor potential cation channel, subfamily C, member 3”	5.5	7.6	4.4	0
*CfaAff × 0.16161.1.S1_s_at	TRPM1*	“transient receptor potential cation channel, subfamily M, member 1”	8.0	9.8	4.6	0
**Neuronal Development**						
*Cfa.9271.1.A1_at	CA10*	carbonic anhydrase X	7.3	10.0	6.1	0
Cfa.10284.1.S1_s_at	CRMP1	collapsin response mediator protein 1	6.1	7.1	2.1	0
CfaAff × 0.16492.1.S1_at	DNER	delta/notch-like EGF repeat containing	8.8	9.7	2.0	0
Cfa.19360.1.S1_s_at	GNAO1	“guanine nucleotide binding protein (G protein), alpha activating activity polypeptide O”	8.9	10.8	3.8	0
Cfa.7236.1.A1_s_at	NRXN1	neurexin 1	7.1	9.4	4.2	0
*Cfa.10306.1.S1_at	OLFM1*	olfactomedin 1	8.6	10.0	2.5	0
CfaAff × 0.30602.1.S1_at	OLFM3	olfactomedin 3	5.8	7.1	2.9	0
*Cfa.9934.1.A1_at	PCP4*	Purkinje cell protein 4	9.6	13.0	8.9	0
Cfa.15689.1.A1_at	PHYH	phytanoyl-CoA 2-hydroxylase	7.1	9.2	4.3	0
CfaAff × 0.12766.1.S1_at	ROBO2	“roundabout, axon guidance receptor, homolog 2”	5.8	7.8	4.2	0
*CfaAff × 0.22493.1.S1_s_at	SERPINI1*	“serpin peptidase inhibitor, clade I, member 1”	10.0	11.8	3.0	0
*Cfa.10644.1.A1_at	SH3GL3*	SH3-domain GRB2-like 3	6.8	9.0	5.2	0
*Cfa.1224.1.S1_at	TAGLN3*	transgelin 3	9.2	11.9	4.9	0
Cfa.174.1.S1_s_at	TFAP2B	transcription factor AP-2 beta	6.9	9.6	4.4	0
**Transcription Factors**						
CfaAff × 0.12012.1.S1_at	GTF2H3	“general transcription factor IIH, polypeptide 3, 34kDa”	5.8	6.8	2.0	0.126704939
CfaAff × 0.28228.1.S1_at	ISL1	ISL LIM homeobox 1	6.2	7.6	2.9	0
CfaAff × 0.5793.1.S1_s_at	MYT1L	myelin transcription factor 1-like	5.5	6.8	2.4	0
CfaAff × 0.25541.1.S1_s_at	RORA	RAR-related orphan receptor A	5.6	6.8	2.1	0.064323389
Cfa.5127.1.A1_s_at	RUNX1T1	runt-related transcription factor 1	6.6	8.1	2.8	0
*CfaAff × 0.20645.1.S1_at	RXRG*	“retinoid X receptor, gamma”	7.0	8.1	2.0	0
**G-protein coupled receptor protein signaling**						
Cfa.11087.1.A1_s_at	BAI3	brain-specific angiogenesis inhibitor 3	7.2	8.9	3.3	0
*CfaAff × 0.8825.1.S1_s_at	CCK*	cholecystokinin	5.4	7.2	2.7	0.126704939
Cfa.3870.1.A1_at	GAL	galanin prepropeptide	5.6	8.0	5.5	0
*Cfa.3177.1.S1_at	GNAI1*	“guanine nucleotide binding protein (G protein), alpha inhibiting activity polypeptide 1”	9.8	11.2	2.8	0
*Cfa.10270.2.A1_at	GNG3*	“guanine nucleotide binding protein (G protein), gamma 3”	7.5	9.5	3.6	0
CfaAff × 0.5919.1.S1_at	GPR85	G protein-coupled receptor 85	7.0	9.0	4.2	0
CfaAff × 0.4269.1.S1_at	NXPH1	neurexophilin 1	6.2	8.9	5.4	0
*Cfa.9688.1.A1_at	NXPH2*	neurexophilin 2	5.8	7.2	2.6	0
Cfa.1416.1.A1_at	PENK	proenkephalin	5.7	7.4	3.2	0
Cfa.11233.1.A1_at	SSTR2	somatostatin receptor 2	5.9	7.0	2.3	0.064323389
**Cytoskeleton**						
CfaAff × 0.5843.1.S1_s_at	ELMO1	engulfment and cell motility 1	6.8	8.8	5.0	0
Cfa.6416.1.A1_at	SGCG	“sarcoglycan, gamma”	6.0	7.0	2.1	0
*Cfa.11292.1.A1_at	TMOD1*	tropomodulin 1	6.9	8.8	3.0	0
*Cfa.10164.1.S1_at	TUBA4A*	“tubulin, alpha 4a”	7.1	9.8	4.9	0
*Cfa.11276.1.S1_s_at	TUBB2A*	“tubulin, beta 2A”	11.1	12.9	3.3	0

Asterisks indicate that elevated decreased levels for this gene were detected by more than one probe set. Expression levels and fold change values are given as log^2^ units.

**Table 4:** Selected genes detected at increased levels in the glaucomatous canine retina.

**Probe Set ID**	**Gene**	**Annotation**	**Glau_avg**	**Cont_avg**	**Fold change**	**q-value (%)**
**Complement components**						
*Cfa.1379.1.S1_at	C1QA*	“complement component 1, q subcomponent, A chain”	11.8	9.6	4.3	0
Cfa.16857.1.S1_at	C1QB	“complement component 1, q subcomponent, B chain”	10.9	8.1	5.5	0
*Cfa.10921.1.S1_at	C1QC*	“complement component 1, q subcomponent, C chain”	11.4	8.0	9.3	0
*Cfa.21168.1.S1_s_at	C1R*	“complement component 1, r subcomponent”	8.0	6.3	2.9	0
*Cfa.10821.1.A1_s_at	C1S*	“complement component 1, s subcomponent”	12.3	10.5	3.5	0
*Cfa.12240.1.A1_at	C3*	complement component 3	13.7	9.1	21.8	0
CfaAff × 0.21480.1.S1_at	C3AR1*	complement component 3a receptor 1	6.9	5.6	2.0	0
*Cfa.14267.1.S1_at	CFB*	complement factor B	11.0	9.3	2.9	0
*Cfa.14495.2.S1_at	CFI*	complement factor I	10.6	8.8	3.2	0
*Cfa.21548.1.S1_s_at	LOC481722*	similar to Complement C4 precursor	8.6	6.3	4.7	0
*Cfa.3117.1.S1_at	SERPING1*	“serpin peptidase inhibitor, clade G (C1 inhibitor)”	12.5	10.3	4.2	0
CfaAff × 0.24815.1.S1_at	TLR1	toll-like receptor 1	7.7	6.5	2.5	0.181332902
CfaAff × 0.11983.1.S1_at	TLR3	toll-like receptor 3	7.9	6.4	2.2	0.181332902
CfaAff × 0.18172.1.S1_at	TLR7	Toll-like receptor 7	7.1	5.8	2.4	0
**Acute Phase Proteins**						
CfaAff × 0.23392.1.S1_x_at	SAA	serum amyloid A protein	7.2	5.8	3.8	0
*Cfa.3173.2.A1_a_at	SAA1*	serum amyloid A1	7.9	4.8	20.1	0
*Cfa.3173.2.A1_a_at	SAA2*	serum amyloid A2	7.9	4.8	20.1	0
Cfa.5989.1.A1_s_at	SERPINA1	“serpin peptidase inhibitor, clade A (alpha-1 antiproteinase, antitrypsin), member 1”	7.9	6.5	2.2	0
*Cfa.14503.1.A1_at	SERPINA3*	“serpin peptidase inhibitor, clade A (alpha-1 antiproteinase, antitrypsin), member 3”	9.7	7.2	5.7	0
*Cfa.5199.1.A1_at	STAT3*	signal transducer and activator of transcription 3	9.6	8.3	2.4	0
**Apoptosis**						
CfaAff × 0.7867.1.S1_s_at	APOE	apolipoprotein E	11.8	9.5	4.4	0
Cfa.110.1.S1_s_at	BCL2	B-cell CLL/lymphoma 2	9.1	7.7	2.6	0
*Cfa.21056.1.S1_at	BCL2A1*	BCL2-related protein A1	8.3	6.2	5.4	0
Cfa.3589.1.S1_s_at	CASP4	“caspase 4, apoptosis-related cysteine peptidase”	8.4	6.3	3.7	0
Cfa.3851.1.S1_s_at	CCL2	chemokine (C-C motif) ligand 2	7.8	6.2	4.7	0
CfaAff × 0.29633.1.S1_s_at	GADD45B	“growth arrest and DNA-damage-inducible, beta”	9.1	7.3	3.2	0
CfaAff × 0.16351.1.S1_s_at	IFIH1	interferon induced with helicase C domain 1	7.9	6.7	2.3	0
Cfa.40.1.S1_s_at	IL18	interleukin 18 (interferon-gamma-inducing factor)	6.9	5.5	2.5	0
Cfa.13715.1.A1_at	LOC479458	similar to caspase 12 (mouse)	7.0	4.8	4.4	0
CfaAff × 0.15920.1.S1_s_at	MX1	“myxovirus (influenza virus) resistance 1, interferon-inducible protein p78 (mouse)”	11.0	9.7	2.2	0
CfaAff × 0.23506.1.S1_at	NTF3	neurotrophin 3	7.2	5.9	3.3	0
*Cfa.9240.1.S1_at	SPP1*	secreted phosphoprotein 1 (osteopontin)	9.7	6.3	9.2	0
*Cfa.18084.1.S1_s_at	STAT1*	signal transducer and activator of transcription 1	7.8	6.2	3.0	0
Cfa.18359.1.S1_at	STK17B	serine/threonine kinase 17b	7.9	6.9	2.2	0
*CfaAff × 0.12369.1.S1_s_at	SULF1*	sulfatase 1	8.8	7.5	2.1	0
Cfa.6225.1.A1_at	TNFRSF14	“tumor necrosis factor receptor superfamily, member 14”	8.3	7.1	2.2	0
CfaAff × 0.23380.1.S1_s_at	TNFRSF1A	“tumor necrosis factor receptor superfamily, member 1A”	7.4	6.3	2.1	0
**Protein degradation**						
*CfaAff × 0.14173.1.S1_s_at	CTSC*	cathepsin C	11.4	8.2	7.9	0
*Cfa.2521.1.S1_at	CTSH*	cathepsin H	12.0	10.0	3.5	0
*Cfa.1661.1.S1_at	CTSS*	cathepsin S	11.5	9.1	4.6	0
CfaAff × 0.18934.1.S1_at	CTSZ	cathepsin Z	8.3	6.9	2.4	0
Cfa.4392.1.S1_at	DNASE2	“deoxyribonuclease II, lysosomal”	9.8	8.5	2.2	0
Cfa.10080.1.A1_at	LAMP2	lysosomal-associated membrane protein 2	9.8	8.5	2.0	0.181332902
*Cfa.9004.1.S1_at	LAPTM5*	lysosomal associated multispanning membrane protein 5	10.4	8.3	3.9	0
*Cfa.11935.1.A1_at	LGMN*	legumain	9.1	8.1	2.1	0
*Cfa.797.1.S1_at	LGALS3	“lectin, galactoside-binding, soluble, 3”	11.9	10.5	2.4	0
CfaAff × 0.9252.1.S1_at	LGALS3BP	“lectin, galactoside-binding, soluble, 3 binding protein”	7.9	6.7	2.3	0
*Cfa.15305.1.S1_at	LYZ*	lysozyme	10.5	7.9	5.2	0
CfaAff × 0.7537.1.S1_at	PRSS23	“protease, serine, 23”	9.3	8.1	2.1	0
*Cfa.12298.1.A1_a_at	PSMB8*	“proteasome subunit, beta type, 8”	8.9	7.2	3.6	0
**Antigen processing/presentation**						
CfaAff × 0.21053.1.S1_s_at	B2M	beta-2-microglobulin	13.1	11.9	2.2	0
*Cfa.20996.1.S1_at	DLA-12*	MHC class I DLA-12	12.2	10.3	3.7	0
CfaAff × 0.1704.1.S1_s_at	DLA-64	MHC class I DLA-64	8.7	6.2	5.6	0
*Cfa.14528.1.A1_at	DLA-79*	MHC class Ib	10.2	8.3	3.8	0
*Cfa.280.1.S1_s_at	dla88*	MHC class I DLA-88	13.6	12.2	2.6	0
*Cfa.182.1.S1_s_at	DLA-DQA1*	“major histocompatibility complex, class II, DQ alpha 1”	7.7	5.4	5.1	0
CfaAff × 0.2152.1.S1_s_at	DLA-DQB1	“major histocompatibility complex, class II, DQ beta 1”	10.4	7.3	9.0	0
*Cfa.6456.1.S1_at	DLA-DRA1*	MHC class II DR alpha chain	12.0	9.3	6.2	0
Cfa.181.1.S1_at	DLA-DRB1	MHC class II DLA DRB1 beta chain	12.3	9.5	6.1	0
Cfa.173.1.A1_s_at	FCGR1A	“Fc fragment of IgG, high affinity Ia, receptor (CD64)”	8.9	6.6	4.5	0
*Cfa.21258.1.S1_at	FCGR3A*	“Fc fragment of IgG, low affinity IIIa, receptor (CD16a)”	8.0	5.2	6.9	0
*Cfa.17806.1.S1_at	FCGRT*	“Fc fragment of IgG, receptor, transporter, alpha”	9.9	8.7	2.3	0
*Cfa.18297.1.S1_at	HLA-DMA*	“major histocompatibility complex, class II, DM alpha”	9.2	7.2	3.9	0
**Inflammation**						
CfaAff × 0.11797.1.S1_s_at	ACOX2	“acyl-Coenzyme A oxidase 2, branched chain”	8.7	7.0	2.7	0
Cfa.14366.1.S1_s_at	AIF1	allograft inflammatory factor 1	8.6	7.3	2.4	0
*Cfa.10210.1.S1_at	ALOX5AP*	arachidonate 5-lipoxygenase-activating protein	9.7	8.1	2.8	0
*Cfa.19174.1.S1_s_at	CD163*	CD163 molecule	6.4	5.0	2.2	0
*Cfa.6017.1.S1_at	CYBB*	“cytochrome b-245, beta polypeptide”	8.4	6.0	5.3	0
Cfa.3634.1.S1_at	ITGB2	“integrin, beta 2 (complement component 3 receptor 3 and 4 subunit)”	8.1	6.8	2.2	0
Cfa.12422.1.A1_at	MGST2	microsomal glutathione S-transferase 2	6.5	5.3	2.5	0
CfaAff × 0.9427.1.S1_at	TNFAIP6	“tumor necrosis factor, alpha-induced protein 6”	10.5	9.0	2.8	0
*CfaAff × 0.3283.1.S1_s_at	TREM2*	triggering receptor expressed on myeloid cells 2	7.8	6.4	2.8	0
**T and B cell proteins**						
*Cfa.14560.1.S1_at	CD48*	CD48 molecule	7.8	5.9	4.0	0
Cfa.3629.2.S1_s_at	CD86	CD86 molecule	9.9	8.0	3.8	0
Cfa.12433.1.A1_at	CD99	CD99 molecule	10.2	8.9	2.0	0
CfaAff × 0.20171.1.S1_s_at	FCRLA	Fc receptor-like A	8.5	5.3	8.4	0
CfaAff × 0.11449.1.S1_at	ID1	inhibitor of DNA binding 1	8.9	7.9	2.1	0
*Cfa.64.1.S1_at	ID3*	inhibitor of DNA binding 3	10.3	8.7	2.9	0
Cfa.4556.2.S1_s_at	IGHAC	IgA heavy chain constant region	7.1	5.6	3.7	0.181332902
Cfa.4556.3.A1_s_at	LOC607467	Ig heavy chain V-III region VH26 precursor	7.9	5.8	5.7	0.181332902
Cfa.15473.1.A1_at	LY86	lymphocyte antigen 86	7.8	6.6	2.0	0
*Cfa.19790.1.S1_at	NFAT5*	“nuclear factor of activated T-cells 5, tonicity-responsive”	9.3	8.2	2.1	0
Cfa.17809.1.S1_s_at	PTPRC	“protein tyrosine phosphatase, receptor type, C”	6.3	5.0	2.4	0
Cfa.11351.1.A1_at	TCIRG1	“T-cell, immune regulator 1, ATPase, H+ transporting, lysosomal V0 subunit A3”	8.7	7.5	2.2	0
**Growth Factors, growth factor binding proteins, and their receptors**						
*Cfa.17361.1.S1_s_at	CSF1R*	colony stimulating factor 1 receptor	9.6	6.4	11.1	0
CfaAff × 0.9754.1.S1_s_at	LTBP1	latent transforming growth factor beta binding protein 1	7.3	5.9	2.5	0
Cfa.18699.1.S1_s_at	LTBP3	latent transforming growth factor beta binding protein 3	8.5	7.3	2.2	0
Cfa.10765.1.S1_s_at	PDGFRA	“platelet-derived growth factor receptor, alpha polypeptide”	8.4	7.3	2.2	0
CfaAff × 0.2176.1.S1_at	TNFRSF11B	“tumor necrosis factor receptor superfamily, member 11b”	8.0	6.4	2.3	0
**Chemokine ligands and receptors**						
CfaAff × 0.21302.1.S1_s_at	CCR5	chemokine (C-C motif) receptor 5	7.4	5.9	3.4	0.181332902
*Cfa.16590.1.S1_s_at	CXCL10*	chemokine (C-X-C motif) ligand 10	6.9	4.7	9.5	0.181332902
*Cfa.20779.1.S1_at	CXCL12*	chemokine (C-X-C motif) ligand 12	8.5	7.1	4.0	0
CfaAff × 0.24352.1.S1_at	CXCL16	chemokine (C-X-C motif) ligand 16	8.0	5.9	4.7	0
Cfa.14516.1.S1_at	IL18BP	interleukin 18 binding protein	9.4	7.8	3.4	0
**Extracellular Matrix Constituents**						
Cfa.10374.1.A1_at	COL12A1	“collagen, type XII, alpha 1”	6.8	4.8	4.2	0
*Cfa.6936.1.A1_at	COMP*	cartilage oligomeric matrix protein	7.7	6.6	2.5	0
*Cfa.15083.1.S1_at	EFEMP1*	fibulin 3	8.7	7.6	2.0	0
*Cfa.4189.1.A1_at	EFEMP2*	fibulin 4	11.4	10.2	2.2	0
*Cfa.19661.1.S1_s_at	FBLN5*	fibulin 5	8.0	6.5	2.2	0
*Cfa.5998.1.A1_x_at	FGG*	fibrinogen gamma chain	8.0	6.7	3.4	0
CfaAff × 0.1090.1.S1_at	MMP19	matrix metallopeptidase 19	7.1	5.6	2.3	0
Cfa.19828.1.S1_at	LOC475881	Neuronal cell adhesion molecule precursor (Nr-CAM)	9.5	8.5	2.0	0
**Misc. Molecules**						
CfaAff × 0.20132.1.S1_at	ACE	angiotensin I converting enzyme 1	9.7	7.4	4.7	0
Cfa.3982.1.A1_at	AGT	angiotensinogen	8.0	6.6	3.2	0
*Cfa.2878.1.A1_s_at	CP*	ceruloplasmin (ferroxidase)	11.0	7.6	10.4	0
*Cfa.19821.1.S1_s_at	GFAP*	glial fibrillary acidic protein	7.0	5.2	3.3	0
CfaAff × 0.9311.1.S1_s_at	GMFG	“glia maturation factor, gamma”	9.2	7.9	2.5	0
Cfa.19828.1.S1_at	LOC475881	similar to Neuronal cell adhesion molecule precursor (Nr-CAM)	9.5	8.5	2.0	0
*Cfa.10277.1.S1_at	S100A1*	S100 calcium binding protein A1	10.9	9.6	2.3	0

Asterisks indicate that elevated levels for this gene were detected by more than one probe set. Expression levels and fold change values are given as log^2^ units.

**Figure 2:**
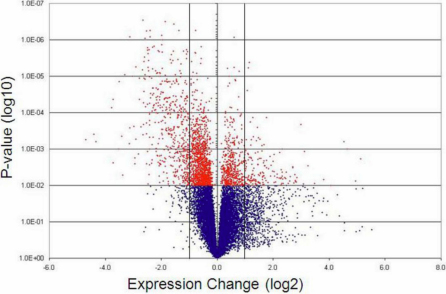
Volcano plot of the gene expression changes in the canine glaucomatous retina. Negative expression changes denote transcripts detected at reduced levels in glaucomatous eyes. This plot also represents all expressed genes, including those with less than twofold expression changes. Vertical bars represent a twofold expression change. Probability values were derived by Student’s *t*-test.

Interestingly a plot of the average detected gene expression changes versus the p-value associated with each measurement reveals a marked asymmetry between transcripts with reduced expression and those with elevated expression (Figure 2). While transcripts detected at lower levels in the glaucomatous retina show a good correlation between the degree of expression change and associated p-value, genes with higher average expression in glaucoma are frequently associated with non-significant p-values. This is true even for genes with comparatively large changes in average expression levels. These higher p-values are typically the result of a large standard deviation between the values in the glaucoma group. These data indicate that the factors causing lower gene expression are largely similar between the individuals evaluated, whereas glaucomatous events resulting in elevated transcript levels are much less uniform.

**Figure 3:**
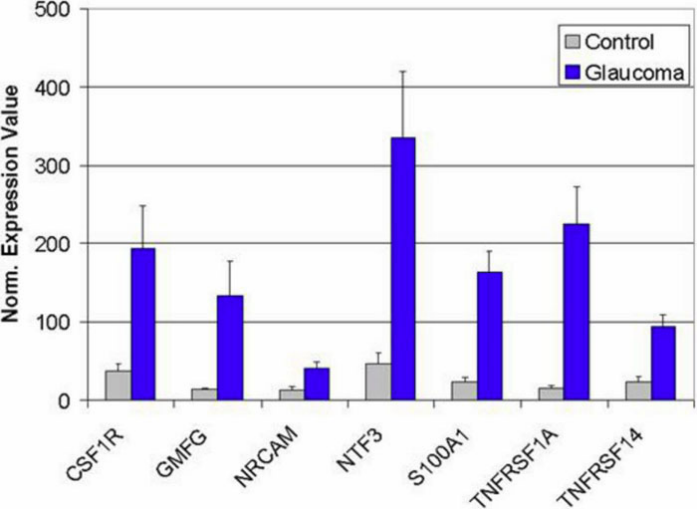
Verification of selected expression changes by quantitative PCR. Elevated expression could be confirmed for colony stimulating factor 1 receptor (*CSF1R*), glia maturation factor gamma (*GMFG*), neuronal cell adhesion molecule (*NRCAM*), neurotrophin3 (*NTR3*), Calgizzarin (*S100A1*), TNF receptor 1 (*TNFRSF1A*), and TNF receptor 14 (*TNFRSF14*). Error bars signify standard error.

To test the accuracy and reproducibility of the Gene chip data we also sought to confirm the observed gene expression differences between glaucomatous and normal retinas using quantitative PCR. Clearly, verification of all gene changes is impractical and consequently only a subset of genes was selected. In the selection of these genes we avoided those with already well described glaucoma related expression changes, such as *GFAP* and complement components [8-12], and focused instead on less well characterized genes (Figure 3). Our RT–PCR data indicate that expression levels vary considerably among the nine affected animals evaluated for this part of the study. However, statistically significantly elevated (p<0.05 by *t*-test) levels of colony stimulating factor 1 receptor (*CSF1R*), glia maturation factor gamma (*GMFG*), neuronal cell adhesion molecule (*NRCAM*), neurotrophin 3 (*NTF3*), Calgizzarin (*S100A1*), TNF receptor 1 (*TNFRSF1A*) and TNF receptor 14 (*TNFRSF14*) were confirmed.

### Immunohistochemical evaluation

**Figure 4:**
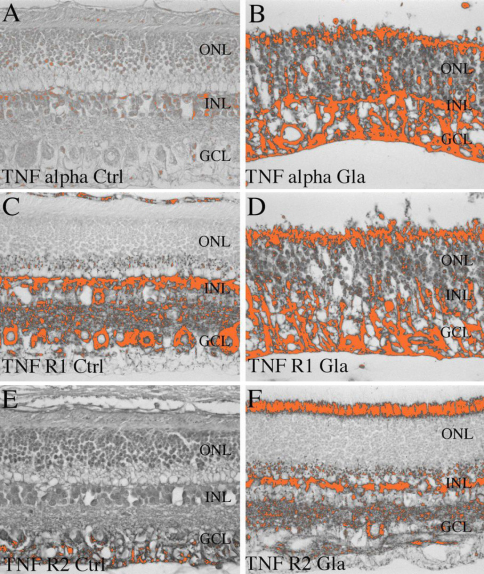
Digitalized images of immunohistochemistry based protein expression, which were used for quantification purposes. Increased TNF alpha expression was detected in glaucomatous eyes (**B**), predominantly in the nerve fiber layer, when compared to the control eyes (**A**). TNF alpha receptor 1 protein expression had similar appearance in control and glaucomatous eyes (**C**, **D**). TNF alpha receptor 2 protein expression was higher in glaucomatous eyes (**F**) when compared to control eyes (**E**).

**Figure 5:**
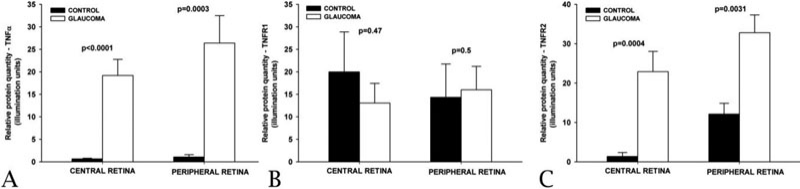
Quantitative analysis of TNF, TNFR1, and TNFR2 expression in the glaucomatous retina. Quantitative analysis of TNFa (**A**), TNF receptor 1 (**B**), and TNF receptor 2 (**C**) immune reactivity in the central and peripheral retina of dogs with and without glaucoma. Statistical analyses reveal significantly higher TNF-alpha (**A**) and TNF-alpha receptor 2 (**C**) expression in glaucomatous eyes, when compared to control eyes. There was no significant difference in TNF alpha receptor 2 expression between control and glaucomatous eyes (**B**).

Our molecular findings, in accord with previously published studies, suggest that modulation of TNF alpha and its receptors is correlated to the development of glaucoma. Morphometric analyses performed on retinas from of healthy and glaucomatous dogs using anti-TNF antibodies demonstrated increased immunereactivity in the glaucomatous retina (Figure 4A,B). Quantitation of the observed signal demonstrated a significant increase in TNF labeling both in the central and the peripheral retina (Figure 5A).

Similar changes were observed for TNF receptor 2 (TNFR2). Immunoreactivity for this molecule is significantly increased in the peripheral and central retina of glaucomatous eyes when compared to normal eyes (Figure 4E,F and Figure 5C). In contrast, our data suggest that expression levels for TNF receptor 1 (TNFR1) remain unchanged in the glaucomatous retina (Figure 4C,D and Figure 5B). The finding that overall TNFR1 immunoreactivity remains relatively unchanged contrasts with our data suggesting elevated mRNA levels of the TNFR1 gene (*TNFRSF1A*) in glaucoma.

**Figure 6:**
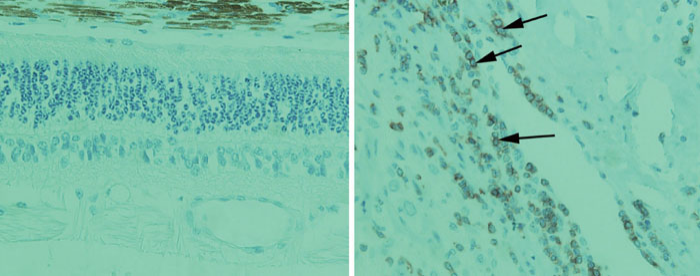
Immunohistochemical detection of CD3. CD3 positive cells are not apparent in the canine glaucomatous retina (left). In contrast, CD3 positive cells can easily be detected in optic nerve sections of a canine patient with optic neuritis (positive control). Note labeled cells in the perivascular space of the optic nerve (arrows). ONL-outer nuclear layer, INL-inner nuclear layer, NFL – nerve fiber layer.

The pronounced appearance of MHC class I and inflammation related gene transcripts could be interpreted as indicative of leukocyte infiltration into the canine glaucomatous retina. Immunohistochemical analyses using sagittal sections of several canine glaucomatous retinas and antibodies directed against the T- and B- cell antigens CD3 and CD79 did not reveal immunopositive cells in the evaluated samples (Figure 6A). CD3 positive cells could readily be detected in a tissue sample of canine optic neuritis, used here as a positive control (Figure 6B), and were occasionally observed in the perivascular space in glaucoma retinas. These findings suggest that leukocyte infiltration into the retina of glaucomatous dogs it is, at most, a rare event.

### Detection of serum autoantibodies to retinal antigens

**Figure 7:**
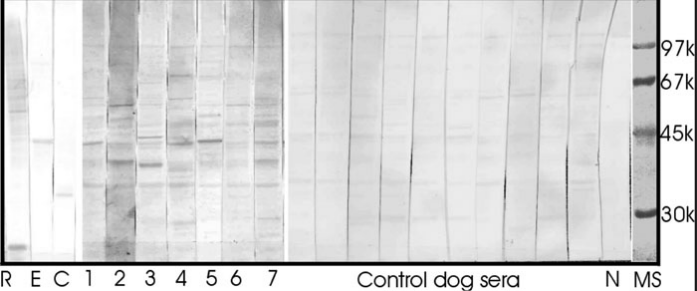
Western blotting analysis of autoantibodies present in sera of dogs with glaucoma and in sera of control healthy dog sera. Sera from dogs with glaucoma are labeled from 1 to 7; control dog sera are labeled as “control dog sera,” Lanes R- recoverin, E – enolase, C - crystalline-μ are immunostained with antibodies specifically directed against these molecules. N – negative control, M – molecular weight markers.

Despite the apparent paucity of CD3/CD79 positive cells, it was conceivable that canine glaucoma may result in the formation of autoantibodies directed against retinal antigens. To determine if this does indeed occur, we incubated western blots of retinal protein extracts with serum obtained from seven dogs with moderate to advanced glaucoma and ten healthy control animals (Figure 7). While minor immune reactivity can be observed in all samples, the majority of the serum obtained from dogs with glaucoma reacts with retinal proteins more vigorously than that obtained from healthy control dogs. It is noteworthy that the samples with the most pronounced immunoreactivity were derived from dogs with advanced glaucoma. A general pattern of labeled bands is not apparent, rather it appears that each individual displays immunereactivity to a specific subset of molecules. In the majority of cases the labeled molecules do not react with purified Recoverin, α-enolase, or crystallins, suggesting that these proteins are not major autoantigens in dogs with glaucoma.

## Discussion

In this study we characterize the molecular events that are associated with advanced glaucomatous degeneration of the retina in dogs. These data are the first to describe changes in the gene expression pattern and immunological consequences in a species with large eyes and spontaneously occurring chronic elevation of IOP. One shortcoming of this study is that the control and glaucoma groups were are not closely matched in age and are derived from a variety of breeds. These factors will likely influence the expression levels of some genes, but the overall similarity in canine retinal morphology and function suggest that these differences between the two groups will be relatively minor.

In accordance with previous studies, our data demonstrate that advanced glaucomatous retinopathy is associated with pronounced changes in the retinal gene expression pattern. Among genes with elevated expression levels in glaucoma, a preponderance of genes mediating various aspects of a neuroinflammatory response was striking. Prominent functional categories of genes with elevated expression in glaucoma include antigen presentation, complement activation, lysosomal and proteasome activity, and acute phase proteins. In addition, numerous genes with a function in apoptosis and inflammation signaling are more abundant in retinas with glaucoma than in those without. It must be noted that many of the identified genes with altered expression levels are associated with several biologic function, thus inclusion in one functional category does not exclude a gene’s involvement in additional molecular pathways.

Our analyses also indicate that glaucoma leads to lower expression levels for a large number of genes, including various neurofilaments, synucleins, and neuregulin 1. Functionally, no particular group of molecules appears to be disproportionally affected. Rather, we detected decreased expression levels of a large number of functional categories, including neuronal development and maintenance, cell adhesion, calcium transport and binding, transcriptional regulation, synaptic transmission, and many others. Reduced mRNA levels of individual genes could result from transcriptional control mechanisms. Alternatively, reduced levels of a specific mRNA in a tissue comprised from several cell types may be related to loss of the cell type that synthesizes the majority of this mRNA. Genes such as *STMN2*, *NEFH*, *NRG1*, *ELAV2*, and *SNCG* appear to be primarily expressed by RGC [13-15] and the decrease of their transcript levels is conceivably due to the loss of RGC and, perhaps, amacrine cells [16,17] in the glaucomatous retina and may not represent transcriptional regulation.

Decreased expression levels were also detected for several photoreceptor cell specific genes in the glaucomatous retina. Whether photoreceptor cell loss or functional decline is a feature of advanced glaucoma has been extensively debated [18-21]. In this study significantly decreased expression levels are only observed for a small number of photoreceptor cell specific genes, while most of photoreceptor cell associated transcripts are present at similar or only mildly reduced levels (i.e., less than the twofold cut off value employed) suggesting only a minor effect of glaucoma on the transcriptional activity of photoreceptor cells in the dog.

The systematic comparison of findings in this study to those presented previously by other investigators is not straightforward due to the different formats of gene arrays used, selection of genes represented on each array, incomplete identification of orthologs between animal species, and the stage of disease investigated. Previous studies had observed a striking decrease in the expression levels for several crystallin genes in rodent models of glaucoma [3-5,22]. Although probes for these molecules are present on the gene chips used in this study, our data do not mirror these findings. Reduced synthesis of retinal crystallins could represent a rodent specific response to elevated IOP. However, several studies have demonstrated that expression levels for various crystallin genes decrease quickly after induction of ocular hypertension, but return to normal levels 2 to 3 weeks later [3,22]. Thus, decreased expression of crystallins may reflect an early, transient event in glaucoma pathology.

Our findings largely agree with previously published reports that clearly demonstrated the involvement of the complement system in the pathophysiology of glaucoma and noted the expression of other inflammation-related molecules by retinal cells [3-5,8,23,24]. While some studies have suggested that a breakdown of the blood brain barrier may occur in the eyes of dogs with severe glaucoma [25,26] our own immunohistochemical data suggest that CD3 or CD79 positive cells in canine glaucoma occur, at most, infrequently. In contrast, most hybridization signals of inflammation associated transcripts are quite strong, indicating that these gene products are abundant in the retina of dogs with glaucoma and thus likely originate from a cell type that is relatively common in this tissue. Micro- and macroglia constitute a sizable share of the retinal cell population and several studies have demonstrated that glia are capable of transcribing several of the inflammation-associated genes detected at elevated levels in this study, e.g., AIF1, CXCL12, and MHC class 2 molecules [27-30]. Retinal glia may also contribute to the observed synthesis of inflammatory molecules, e.g., both CXCL10 and CXCL16 are produced by reactive astrocytes [31,32].

An important function of glia is to phagocytose foreign particles or cell debris, a process activated by the *LGALS3* gene [33]. Macrophages that have ingested apoptotic cells appear to inhibit the production of proinflammatory molecules [34] and is possible that retinal microglia are capable of mediating a similar repression of some proinflammatory stimuli after phagocytosis of RGC debris. In that regard, the increased expression of the *TREM2* receptor in the glaucomatous retina is noteworthy, as its interaction with its ligands appears to mediate inhibition of inflammation and stimulation of antigen presentation [35].

We propose that the massive retinal inflammation and antigen presentation responses described here represent late events in the pathophysiology of glaucoma. Increased expression of individual inflammation-related genes has been reported previously in studies that evaluated earlier stages of glaucomatous damage. While this neuroinflammatory response was not observed to the degree seen here data presented by Ahmed et al. suggest progressively increasing expression levels of several immune response molecules during the development of the disease [3].

Our data suggest that the anti-inflammatory mechanisms that protect the retina in early glaucoma can eventually fail, leading to the development of autoantibodies in many glaucomatous dogs. Although the number of examined samples is relatively small, the fact that all analyzed serum samples from dogs with glaucoma display immunereactivity against distinct antigens, suggests that glaucoma is not initiated by an immune response to specific retinal antigens. Rather it appears likely that the variety of immunoreactive molecules is due to exposure of numerous epitopes during the rapid neuronal cell death of the retina. Consequently the development of anti-retinal antibodies may be secondary to the degeneration of RGC. Never-the-less, exposure to serum antibodies directed against retinal antigens has been shown to induce RGC loss that resembles glaucoma [36,37]. Thus it is conceivable that once an immune response has occurred, it will further accelerate vision loss in an IOP independent mechanism.

The relevance of our findings to human disease remains to be determined. Glaucoma in dogs often presents with comparatively high intraocular pressure, introducing the possibility that ischemic events to the inner retina contribute to the pathophysiology of glaucoma in this species more than they do in humans. It is well documented that retinal ischemia leads to increased vascular permeability (reviewed in [38]) and given the chronic nature of IOP elevation in glaucoma it is conceivable that even a rare presence of T-cells could over time result in an antigenic response in this species. The intraocular pressure in human patients receiving ophthalmic care is typically much lower than that in glaucomatous dogs and leukocyte infiltration of the retina does not appear to be a feature of human glaucoma. Yet several reports have suggested the presence of autoantibodies against retinal epitopes in the serum of some glaucoma patients [39-41]. Whether those glaucoma patients with detectable autoimmune titers to the retina share a genetic predisposition toward a vigorous immune response or exhibit other unifying features has, to our knowledge, not been thoroughly investigated.

Regardless of the potential role of an adaptive immune response in glaucoma, data from numerous studies indicate that neuroinflammatory events do occur as a consequence of neuronal degeneration in glaucoma. Consequently it is conceivable that therapeutic modulation of the neuroinflammatory response may be a beneficial augmentation to IOP lowering therapy in glaucoma.

## References

[1] Grozdanic SD Matic M Betts DM Sakaguchi DS Kardon RH Recovery of canine retina and optic nerve function after acute elevation of intraocular pressure: implications for canine glaucoma treatment. Vet Ophthalmol 2007 10 101 7 17973841 10.1111/j.1463-5224.2007.00584.x

[2] Grozdanic S Kecova H Harper MM Nilaweera WU Kuehn MH Kardon RH Functional and structural changes in a canine model of hereditary primary angle-closure glaucoma. Invest Ophthalmol Vis Sci 2010 51 255 63 19661222 10.1167/iovs.09-4081 PMC3258664

[3] Ahmed F Brown KM Stephan DA Morrison JC Johnson EC Tomarev SI Microarray analysis of changes in mRNA levels in the rat retina after experimental elevation of intraocular pressure. Invest Ophthalmol Vis Sci 2004 45 1247 58 15037594 10.1167/iovs.03-1123

[4] Naskar R Thanos S Retinal gene profiling in a hereditary rodent model of elevated intraocular pressure. Mol Vis 2006 12 1199 210 17102796

[5] Steele MR Inman DM Calkins DJ Horner PJ Vetter ML Microarray analysis of retinal gene expression in the DBA/2J model of glaucoma. Invest Ophthalmol Vis Sci 2006 47 977 85 16505032 10.1167/iovs.05-0865

[6] Johnson EC Jia L Cepurna WO Doser TA Morrison JC Global changes in optic nerve head gene expression after exposure to elevated intraocular pressure in a rat glaucoma model. Invest Ophthalmol Vis Sci 2007 48 3161 77 17591886 10.1167/iovs.06-1282 PMC1950563

[7] Yang Z Quigley HA Pease ME Yang Y Qian J Valenta D Zack DJ Changes in Gene Expression in Experimental Glaucoma and Optic Nerve Transection: The Equilibrium between Protective and Detrimental Mechanisms. Invest Ophthalmol Vis Sci 2007 48 5539 48 18055803 10.1167/iovs.07-0542

[8] Kuehn MH Kim CY Ostojic J Bellin M Alward WLM Stone EM Sakaguchi DS Grozdanic SD Kwon YH Retinal synthesis and deposition of complement components induced by ocular hypertension. Exp Eye Res 2006 83 620 8 16677633 10.1016/j.exer.2006.03.002

[9] Stasi K Nagel D Yang X Wang RF Ren L Podos SM Mittag T Danias J Complement Component 1Q (C1Q) Upregulation in Retina of Murine, Primate, and Human Glaucomatous Eyes. Invest Ophthalmol Vis Sci 2006 47 1024 9 16505037 10.1167/iovs.05-0830

[10] Tanihara H Hangai M Sawaguchi S Abe H Kageyama M Nakazawa F Shirasawa E Honda Y Up-regulation of glial fibrillary acidic protein in the retina of primate eyes with experimental glaucoma. Arch Ophthalmol 1997 115 752 6 9194727 10.1001/archopht.1997.01100150754011

[11] Kuehn MH Fingert JH Kwon YH Retinal ganglion cell death in glaucoma: mechanisms and neuroprotective strategies. Ophthalmol Clin North Am 2005 18 383 95 16054996 10.1016/j.ohc.2005.04.002

[12] Woldemussie E Wijono M Ruiz G Muller cell response to laser-induced increase in intraocular pressure in rats. Glia 2004 47 109 19 15185390 10.1002/glia.20000

[13] Kim CY, Kuehn MH, Clark AF, Kwon YH. Laser capture microdissection and microarray analysis of the human retinal ganglion cell layer. ARVO Annual Meeting; 2006 April 30-May 4; Fort Lauderdale (FL).

[14] Surguchov A Palazzo RE Surgucheva I Gamma synuclein: subcellular localization in neuronal and non-neuronal cells and effect on signal transduction. Cell Motil Cytoskeleton 2001 49 218 28 11746666 10.1002/cm.1035

[15] Ivanov D Dvoriantchikova G Nathanson L McKinnon SJ Shestopalov VI Microarray analysis of gene expression in adult retinal ganglion cells. FEBS Lett 2006 580 331 5 16376886 10.1016/j.febslet.2005.12.017

[16] Wang X Ng YK Tay SS Factors contributing to neuronal degeneration in retinas of experimental glaucomatous rats. J Neurosci Res 2005 82 674 89 16273539 10.1002/jnr.20679

[17] Kielczewski JL Pease ME Quigley HA The effect of experimental glaucoma and optic nerve transection on amacrine cells in the rat retina. Invest Ophthalmol Vis Sci 2005 46 3188 96 16123418 10.1167/iovs.05-0321 PMC1236985

[18] Nork TM Ver Hoeve JN Poulsen GL Nickells RW Davis MD Weber AJ Vaegan, Sarks SH, Lemley HL, Millecchia LL. Swelling and loss of photoreceptors in chronic human and experimental glaucomas. Arch Ophthalmol 2000 118 235 45 10676789 10.1001/archopht.118.2.235

[19] Wygnanski T Desatnik H Quigley HA Glovinsky Y Comparison of ganglion cell loss and cone loss in experimental glaucoma. Am J Ophthalmol 1995 120 184 9 7639302 10.1016/s0002-9394(14)72606-6

[20] Holopigian K Greenstein VC Seiple W Hood DC Ritch R Electrophysiologic assessment of photoreceptor function in patients with primary open-angle glaucoma. J Glaucoma 2000 9 163 8 10782626 10.1097/00061198-200004000-00006

[21] Velten IM Korth M Horn FK The a-wave of the dark adapted electroretinogram in glaucomas: are photoreceptors affected? Br J Ophthalmol 2001 85 397 402 11264126 10.1136/bjo.85.4.397 PMC1723933

[22] Piri N Song M Kwong JM Caprioli J Modulation of alpha and beta crystallin expression in rat retinas with ocular hypertension-induced ganglion cell degeneration. Brain Res 2007 1141 1 9 17316577 10.1016/j.brainres.2006.11.095

[23] Miyahara T Kikuchi T Akimoto M Kurokawa T Shibuki H Yoshimura N Gene microarray analysis of experimental glaucomatous retina from cynomologous monkey. Invest Ophthalmol Vis Sci 2003 44 4347 56 14507879 10.1167/iovs.02-1032

[24] Tezel G Yang X Luo C Peng Y Sun SL Sun D Mechanisms of immune system activation in glaucoma: oxidative stress-stimulated antigen presentation by the retina and optic nerve head glia. Invest Ophthalmol Vis Sci 2007 48 705 14 17251469 10.1167/iovs.06-0810 PMC2494942

[25] Mangan BG Al-Yahya K Chen CT Gionfriddo JR Powell CC Dubielzig RR Ehrhart EJ Madl JE Retinal pigment epithelial damage, breakdown of the blood-retinal barrier, and retinal inflammation in dogs with primary glaucoma. Vet Ophthalmol 2007 10 117 24 17973843 10.1111/j.1463-5224.2007.00585.x

[26] Smedes SL Dubielzig RR Early degenerative changes associated with spontaneous glaucoma in dogs. J Vet Diagn Invest 1994 6 259 63 8068762 10.1177/104063879400600221

[27] Liu G Ma H Jiang L Zhao Y Allograft inflammatory factor-1 and its immune regulation. Autoimmunity 2007 40 95 102 17453710 10.1080/08916930601083946

[28] Schmid CD Sautkulis LN Danielson PE Cooper J Hasel KW Hilbush BS Sutcliffe JG Carson MJ Heterogeneous expression of the triggering receptor expressed on myeloid cells-2 on adult murine microglia. J Neurochem 2002 83 1309 20 12472885 10.1046/j.1471-4159.2002.01243.x PMC2637869

[29] Aloisi F Immune function of microglia. Glia 2001 36 165 79 11596125 10.1002/glia.1106

[30] Kaur C Sivakumar V Yip GW Ling EA Expression of syndecan-2 in the amoeboid microglial cells and its involvement in inflammation in the hypoxic developing brain. Glia 2009 57 336 49 18803305 10.1002/glia.20764

[31] Biancotti JC Kumar S de Vellis J Activation of inflammatory response by a combination of growth factors in cuprizone-induced demyelinated brain leads to myelin repair. Neurochem Res 2008 33 2615 28 18661234 10.1007/s11064-008-9792-8

[32] Ludwig A Schulte A Schnack C Hundhausen C Reiss K Brodway N Held-Feindt J Mentlein R Enhanced expression and shedding of the transmembrane chemokine CXCL16 by reactive astrocytes and glioma cells. J Neurochem 2005 93 1293 303 15934948 10.1111/j.1471-4159.2005.03123.x

[33] Rotshenker S The role of Galectin-3/MAC-2 in the activation of the innate-immune function of phagocytosis in microglia in injury and disease. J Mol Neurosci 2009 39 99 103 19253007 10.1007/s12031-009-9186-7

[34] Fadok VA Bratton DL Konowal A Freed PW Westcott JY Henson PM Macrophages that have ingested apoptotic cells in vitro inhibit proinflammatory cytokine production through autocrine/paracrine mechanisms involving TGF-beta, PGE2, and PAF. J Clin Invest 1998 101 890 8 9466984 10.1172/JCI1112 PMC508637

[35] Takahashi K Rochford CD Neumann H Clearance of apoptotic neurons without inflammation by microglial triggering receptor expressed on myeloid cells-2. J Exp Med 2005 201 647 57 15728241 10.1084/jem.20041611 PMC2213053

[36] Joachim SC Grus FH Kraft D White-Farrar K Barnes G Barbeck M Ghanaati S Cao S Li B Wax MB Complex antibody profile changes in an experimental autoimmune glaucoma animal model. Invest Ophthalmol Vis Sci 2009 50 4734 42 19458332 10.1167/iovs.08-3144

[37] Wax MB Tezel G Yang J Peng G Patil RV Agarwal N Sappington RM Calkins DJ Induced autoimmunity to heat shock proteins elicits glaucomatous loss of retinal ganglion cell neurons via activated T-cell-derived fas-ligand. J Neurosci 2008 28 12085 96 19005073 10.1523/JNEUROSCI.3200-08.2008 PMC2683273

[38] Kaur C Foulds WS Ling EA Blood-retinal barrier in hypoxic ischaemic conditions: basic concepts, clinical features and management. Prog Retin Eye Res 2008 27 622 47 18940262 10.1016/j.preteyeres.2008.09.003

[39] Grus FH Joachim SC Bruns K Lackner KJ Pfeiffer N Wax MB Serum autoantibodies to alpha-fodrin are present in glaucoma patients from Germany and the United States. Invest Ophthalmol Vis Sci 2006 47 968 76 16505031 10.1167/iovs.05-0685

[40] Wax M Yang J Tezel G Autoantibodies in glaucoma. Curr Eye Res 2002 25 113 6 12525965 10.1076/ceyr.25.2.113.10157

[41] Tezel G Seigel GM Wax MB Autoantibodies to small heat shock proteins in glaucoma. Invest Ophthalmol Vis Sci 1998 39 2277 87 9804136

